# Association of blood pressure and dietary intake of *Sunomono,* Japanese vinegared side dishes, in community-dwelling Japanese: A cross-sectional study

**DOI:** 10.1016/j.heliyon.2022.e09505

**Published:** 2022-05-21

**Authors:** Hiroaki Kanouchi, Mikako Yamashita, Kaori Kaimoto, Akiko Kuwabara, Yukiko Kawakami, Shigeo Takenaka, Chihaya Koriyama, So Kuwahata, Toshihiro Takenaka, Yuichi Akasaki, Takuro Kubozono, Masaaki Miyata, Mitsuru Ohishi

**Affiliations:** a Department of Clinical Nutrition, Graduate School of Comprehensive Rehabilitation, Osaka Prefecture University, 3–7–30 Habikino, Habikino, Osaka 583-8555, Japan; b Department of Life and Environmental Science, Kagoshima Prefectural College, 1-52-1 Shimoishiki, Kagoshima 890-0005, Japan; c Department of Human Life and Science, Kagoshima Women's College, 6-9 Kohraicho, Kagoshima 890-8565, Japan; d Department of Epidemiology and Preventive Medicine, Kagoshima University Graduate School of Medical and Dental Sciences, 8-35-1 Sakuragaoka, Kagoshima 890-8544, Japan; e Tarumizu Municipal Medical Center, Tarumizu Chuo Hospital, 1-140 Kinkoucho, Tarumizu, Kagoshima 891-2124, Japan; f Department of Cardiovascular Medicine and Hypertension, Graduate School of Medical and Dental Sciences, Kagoshima University, 8-35-1 Sakuragaoka, Kagoshima 890-8544, Japan; g School of Health Sciences, Faculty of Medicine, Kagoshima University, 8-35-1 Sakuragaoka, Kagoshima 890-8544, Japan

**Keywords:** Vinegar, Hypertension, BDHQ, Sea weed

## Abstract

**Objective:**

Vinegar has been reported to have a hypotensive effect. We aimed to investigate the relationship between the consumption of vinegar-based side dishes and blood pressure.

**Research methods & procedures:**

This cross-sectional study included 746 individuals (257 men and 489 women) aged ≥40 years from Tarumizu, Kagoshima, Japan. Nutrient intake was estimated based on the brief-type self-administered diet history questionnaire. The intake frequency of vinegar-based side dishes (*Sunomono* and pickles) was determined using a self-administered diet history questionnaire. Participants who did not consume vinegar-based side dishes for a month were defined as having no *Sunomono* or pickle eating habit. Blood pressure was categorized into four groups according to the Japanese Society of Hypertension Guidelines for the Management of Hypertension. The association between the intake of vinegar-based side dishes and blood pressure categories was analyzed using ordinal logistic regression analysis adjusted for age, body mass index, smoking history, excessive alcohol intake, living situation, energy intake, protein intake, sodium intake, potassium intake, and seaweed intake.

**Results:**

Approximately 13.6% men and 6.1% women had no *Sunomono* eating habits. In men, eating *Sunomono*, but not pickles, was significantly related to blood pressure categories (estimate, −0.702; 95% CI, −1.122 to −0.310), whereas more frequent consumption of *Sunomono* did not show an improvement in the blood pressure category. The relationship between eating *Sunomono* and blood pressure categories was not recognized in women.

**Conclusion:**

This was the first study assessing the association between consumption of vinegar-based side dishes and blood pressure categories. We highlighted the effect of *Sunomono* consumption on blood pressure categories in men. Consumption of *Sunomono* may improve blood pressure in men.

## 1 Introduction

Hypertension is one of the most important public health problems and may be a powerful and modifiable risk factor for cardiovascular diseases. Preventing hypertension requires a healthy diet, moderate exercise, no smoking, and reducing alcohol intake and stress [1]. A healthy diet consists of adequate energy intake to prevent obesity, salt reduction, increased fruit and vegetable intake, cholesterol/saturated fatty acid reduction, and increased polyunsaturated fatty acid and low-fat dairy product intake [2].

The blood pressure-lowering effects of several food-related compounds were well summarized by Venkatakrishnan et al. [3]. The Commissioner of the Consumer Affairs Agency in Japan has approved several foods specifically for health use related to hypertension. These foods have specific functional compounds, such as peptides, Tochu leaf glycoside, acetic acid, γ-aminobutyric, and flavonoids. However, the use of these foods or supplements is not presently recommended in the Japanese Society of Hypertension Guidelines because there are insufficient data regarding their hypotensive effects [2].

Commercially provided vinegar is produced from fruits or cereals via bacterial fermentation. In Asian countries, cereal vinegars are mainly consumed. Vinegar is not only used as a seasoning to add sour flavor but also to preserve food. The health benefits of vinegar have been reported, including anti-diabetic, anti-tumor, anti-obesity, antihypertensive, anti-inflammatory, and cholesterol metabolism-regulating effects [4, 5]. Acetic acid (>4%), the main component of vinegar, has been shown to have antihypertensive effects in animal studies [6]. Na et al. described the mechanism by which acetic acid decreases angiotensin II type 1 receptor expression via the AMPK/PGC-1α/PPARγ pathway [7]. An intervention study addressing the effects of vinegar intake on obesity showed that 30 mL/day of continuous vinegar intake for 12 weeks decreased the body mass index (BMI) from 27.0 kg/m^2^ to 26.3 kg/m^2^ and systolic blood pressure (SBP) from 126 mmHg to 121 mmHg [8]. These changes were significantly different from those observed in the placebo group. It remains unclear whether vinegar has a direct antihypertensive effect or whether blood pressure is lowered as a result of weight loss. The causes of the decrease in SBP because of vinegar intake have not been discussed in detail.

We evaluated whether the consumption of vinegared dishes affected blood pressure. In Japan, major vinegar-based dishes are sliced cucumber-*Sunomono*, Wakame-*Sunomono*, Mozuku-*Sunomono*, and Mekabu-*Sunomono*. *Sunomono* refers to a vinegared dish. Wakame, Mozuku, and Mekabu are seaweed names in Japanese. Pickled vegetables were also consumed. We interviewed participants regarding the frequency of intake of vinegared dishes. We then cross-sectionally evaluated the association between the consumption of vinegar-based dishes and blood pressure.

## 2 Methods

### 2.1 Participants

**Figure 1:**
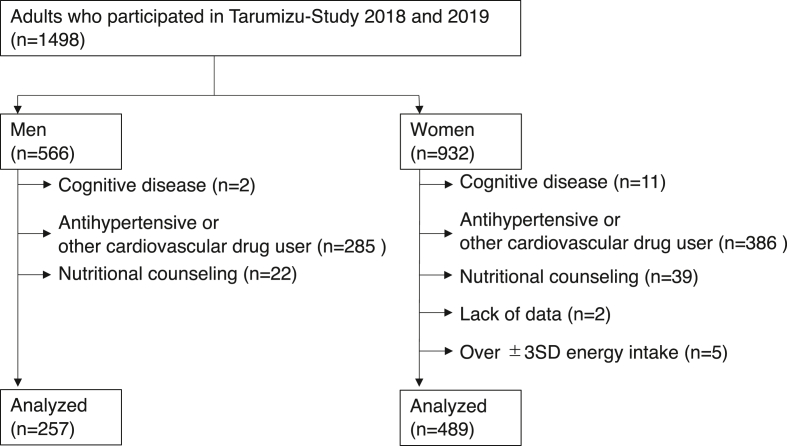
Participant inclusion criteria flow diagram.

This cross-sectional study used data from the Tarumizu Study, 2018 and 2019. The Tarumizu Study is a community-based health check survey that focuses on the health of older people [9]. Individuals selected for participation in the Tarumizu Study were chosen from people (≥40 years) living in Tarumizu City, a local city in Kagoshima, Japan. Participants were recruited through local newspapers and campaigns during community events. A total of 1498 individuals were enrolled in this study (Figure 1). We excluded participants with a history of dementia (n = 13), use of antihypertensive or other cardiovascular drug(s) (n = 671), nutritional counseling (n = 61), lack of data (n = 2), and over ±3 standard deviations (SD) of energy intake (n = 5). Finally, data from 746 people (mean age 66 years; 65.6% women) were analyzed. Informed consent was obtained from all participants before inclusion in the study, and the Ethics Committee of the Faculty of Medicine, Kagoshima University approved the study protocol (No. 170351).

### 2.2 Diet history questionnaire

Dietary habits during the month preceding the intervention were assessed using a previously validated brief-type self-administered diet history questionnaire (BDHQ) [10, 11]. The nutrient intake values were adjusted for energy using the residual method, and a linear regression model was used to minimize the influence of dietary misreporting. The relationship between total dietary fiber and potassium was strongly correlated (r = 0.86, p < 0.0001) according to Pearson's χ^2^-test. This result was attributed to the high potassium content of fiber-rich foods. To avoid multicollinearity, the total dietary fiber values were not used. The frequency of vinegared foods was obtained from the participants using a newly developed self-administered questionnaire (Table 1), and the data were entered into a database using tablet PCs by dieticians. We asked questions regarding vinegared foods distinguished as *Sunomono* (e.g., vinegared cucumber or seaweeds, such as cucumber-*Sunomono*, Wakame-*Sunomono*, Mozuku-*Sunomono*, and Mekabu-*Sunomono*) and pickles (e.g., Japanese pickled scallions). Participants selected the frequency categories, including daily, four to six times per week, two to three times per week, once a week, less than once a week, or not consumed during the past month. Portion size data was not collected.

**Table 1:** Vinegar-related dietary habits questionnaire/consumption frequency.

Sour staples (Sushi, etc.)	Sour main dishes (Sweet and sour pork, etc.)	*Sunomono* (Wakame-su, Mozuku-su, etc.)	Pickles (*Rakkyo*, etc)	Salad with sour dressing
>4 times/mo	>twice/d	>twice/d	>twice/d	>twice/d
4 times/mo	twice/d	twice/d	twice/d	twice/d
3 times/mo	4–6 times/wk	4–6 times/wk	4–6 times/wk	4–6 times/wk
2 times/mo	2–3 times/wk	2–3 times/wk	2–3 times/wk	2–3 times/wk
1 time/mo	1time/wk	1time/wk	1time/wk	1time/wk
never	<1 time/wk	<1 time/wk	<1 time/wk	<1 time/wk
	never	never	never	never

### 2.3 Covariates

Licensed doctors interviewed the participants regarding their medical conditions and history. Nurses measured blood pressure in a seated position using a manual device after the participants were relaxed for more than 5 min. SBP was considered as the blood pressure (BP) at which the first Korotkoff sound was heard and diastolic blood pressure (DBP) was regarded as the BP at which the fifth sound could no longer be heard. Blood pressure was basically measured once, but if the SBP exceeded 180 mmHg, it was measured again for confirmation. Bodyweight was assessed by multifrequency bioelectrical impedance analysis using an Inbody 430 (Inbody Japan, Tokyo, Japan). Participants were asked questions regarding smoking history, education, and living status using a questionnaire on a tablet PC. The questionnaire was previously described in detail [9].

### 2.4 Statistical analysis

Data are presented as medians (interquartile range). The Wilcoxon/Kruskal-Wallis test was used for continuous values to detect significant differences between the two groups. Steel-Dwass test was used for continuous values to detect significant differences among the blood pressure categories. Categorical values were assessed using Pearson's χ^2^-test. Blood pressure levels were categorized into four groups according to the Japanese Society of Hypertension Guidelines for the Management of Hypertension [2]. Group I (normal blood pressure, SBP <120 mmHg or DBP <80 mmHg), Group II (high normal blood pressure, SBP <130 mmHg or DBP <80 mmHg), Group III (elevated blood pressure, SBP <140 mmHg or DBP <90 mmHg), and Group IV (>grade I hypertension, SBP ≥140 mmHg or DBP ≥90 mmHg) are mentioned in Table 3. For each sex, the association between blood pressure categories and intake frequency of *Sunomono* was examined using ordinal logistic regression analyses. Participants consuming more than 20 g/d of alcohol were defined as having excessive alcohol intake. Total energy intake estimated from the BDHQ (energy intake), %energy from protein intake/total energy intake (protein%E), sodium intake, potassium intake, and seaweed intake for men was divided into quartiles as follows: energy intake: <1693, <2124, <2506, and ≥2506 kcal/day, protein%E: <12.8, <14.9, <16.5, and ≥16.5%, sodium intake: <4114, <4566, <5173, and ≥5173 mg/day, potassium intake: <2283, <2770, <3289, and ≥3289 mg/day, and seaweed intake: <2.9, <12.8, <17.1, and ≥17.1 g/day. For women, these values were energy intake: <1345, <1646, <1965, and ≥1965 kcal/day, protein%E intake: <14.8, <16.6, <18.6, and ≥18.6%, sodium intake: <3550, <4044, <4509, and ≥4509 mg/day, potassium intake: <2393, <2725, <3177, and ≥3177 mg/day, and seaweed intake: <4.4, <12.3, <23.4, and ≥23.4 g/d.

The models in the ordinal logistic regression analyses were adjusted for age, BMI, smoking history, living status, alcohol over-consumption (≥20 g/d), energy intake, protein %E, sodium, potassium, and seaweed intake as variables in men. The analysis was conducted in the same manner for women, except for smoking history and alcohol over-consumption (≥20 g/d), because most women who participated in this experiment did not smoke or drink alcohol. Statistical analyses were performed using JMP ver. 14 (SAS Institute Inc., Cary, NC, USA). The significance threshold was set at p < 0.05.

## 3 Results

Significant differences were detected in height, weight, BMI, SBP, and DBP between the sexes (Table 2). Nutrient intake was also different between the sexes, except for potassium intake (Table 2). Men consumed more sodium, saturated fatty acids, and polyunsaturated fatty acids than women. Protein%E, fat%E, and carbohydrate%E were lower in men than in women because alcohol intake was higher in men. The frequency of *Sunomono* consumption among men was 0.4% for twice a day or more, 6.6% once a day, 10.1% four to six times a week, 21.8% two to three times a week, 27.2% once a week, 20.0% less than once a week, and 13.6% never. Among females, 2.2% consumed *Sunomono* more than twice a day, 12.7% once a day, 14.1% four to six times a week, 31.7% two to three times a week, 18.8% once a week, 14.1% less than once a week, and 6.1% never. Because participant characteristics for blood pressure and eating habits differed by sex, further analyses were done conducted by sex.

**Table 2:** Characteristics of participants.

	Men (n = 257)	Women (n = 489)	*p*
Age (y)	66 (58–73)	66 (59–72)	0.719
Height (cm)	159 (151–166)	155 (150–161)	<0.001
Weight (kg)	58.7 (51.1–66.8)	54.5 (49.3–61.9)	<0.001
BMI (kg/m^2^)	23.2 (21.5–25.0)	22.5 (20.8–24.9)	0.045
SBP (mmHg)	135 (123–150)	132 (120–146)	0.017
DBP (mmHg)	81 (74–89)	78 (71–86)	<0.001
Habitual intake of *Sunomono*#	222 (86%)	459 (94%)	<0.001
Habitual intake of pickles	161 (63%)	326 (67%)	0.274
Energy intake (kcal/d)	2124 (1693–2506)	1646 (1345–1964)	<0.001
Protein (%E)	14.9 (12.8–16.5)	16.6 (14.8–18.6)	<0.001
Fat (%E)	25.2 (21.6–29.0)	28.9 (25.8–32.4)	<0.001
Carbohydrate (%E)	50.8 (45.8–56.2)	52.5 (47.9–56.4)	0.017
Sodium (mg/d)	4566 (4114–5173)	4041 (3550–4509)	<0.001
Potassium (mg/d)	2770 (2283–3289)	2725 (2393–3177)	0.925
SFA (g/d)	15.3 (12.9–18.2)	14.6 (12.8–16.6)	0.004
PUFA (g/d)	15.0 (12.9–17.2)	13.8 (12.2–15.4)	<0.001
n-3 PUFA (g/d)	3.0 (2.5–3.7)	2.9 (2.5–3.4)	0.008
Alcohol (g/d)	14.7 (0.5–40.0)	0 (0–0.62)	<0.001

# *Sunomono* is a side dishes containing vinegar. Data were presented as median (interquartile range). The Wilcoxon/Kruskal-Wallis test was used for continuous values to detect significant differences between the two groups. Categorical values were assessed using Pearson's χ2-test. DBP, Diastolic blood pressure; SBP, Systolic blood pressure; BMI, body mass index.

Participants were categorized into four blood pressure categories according to the Japanese Society of Hypertension Guidelines for the Management of Hypertension (Table 3) [3]. Blood pressure categories showed a positive correlation with age. When the relationship between age and blood pressure was evaluated using Pearson's χ^2^-test, age was positively associated with SBP in both men (r = 0.31, p < 0.0001) and women (r = 0.32, p < 0.0001). DBP did not show significant association with age. In women, BMI, education period, and living alone were significantly associated with blood pressure categories. BMI was positively correlated with SBP (r = 0.19, p < 0.001) and DBP (r = 0.38, p < 0.001) using Pearson's χ^2^-test. The percentage of women in a long education period (≥14 years) was high in group I; however, the education period was also strongly related to age. Participants who had cohabitant(s), i.e., lived with others, were associated with increased blood pressure categories in women but not men.

**Table 3:** Characteristics of participants among blood pressure categories.

Blood pressure categories	Men					Women				
	Group I (n = 28, 11%)	Group II (n = 40, 16%)	Group III (n = 78, 30%)	Group VI (n = 111, 43%)	*p*	Group I (n = 108, 22%)	Group II (n = 66, 14%)	Group III (n = 126, 26%)	Group VI (n = 189, 39%)	*p*
SBP DBP	<120 mmHg or <80 mmHg	<130 mmHg or <80 mmHg	<140 mmHg or <90 mmHg	≥140 mmHg or ≥90 mmHg		<120 mmHg or <80 mmHg	<130 mmHg or <80 mmHg	<140 mmHg or <90 mmHg	≥140 mmHg or ≥90 mmHg	
Age (y)	53 (46–65)^a^	67 (59–72)^ab^	65 (59–71)^b^	69 (62–75)^b^	<0.001	59 (50–68)^a^	65 (57–70)^b^	65 (60–72)^bc^	69 (63–74)^c^	<0.001
SBP (mmHg)	112 (110–117)^a^	123 (120–126)^b^	131 (125–138)^c^	153 (145–164)^d^	<0.001	112 (105–116)^a^	123 (121–126)^b^	132 (130–135)^c^	150 (142–160)^d^	<0.001
DBP (mmHg)	70 (67–75)^a^	73 (68–76)^a^	82 (80–86)^b^	90 (83–95)^c^	<0.001	69 (63–72)^a^	74 (70–77)^b^	81 (75–84)^c^	89 (80–94)^d^	<0.001
BMI (kg/m^2^)	22.5 (26.2)	22.8 (21.3–24.5)	23.4 (21.8–24.8)	23.0 (21.5–25.4)	0.738	21.6 (20.1–24.0)^a^	22.3 (20.8–24.3)^ab^	22.7 (21.3–24.7)^ab^	23.2 (21.0–25.7)^b^	0.001
Smoking n (%) Never	7 (25%)	10 (25%)	19 (24%)	33 (30%)		91 (84%)	58 (88%)	116 (92%)	180 (95%)	
Former	11 (39%)	23 (58%)	43 (55%)	57 (51%)	0.569	12 (11%)	8 (12%)	7 (6%)	5 (3%)	0.009
Current	10 (36%)	7 (18%)	16 (21%)	21 (19%)		5 (5%)	0 (0%)	3 (2%)	4 (2%)	
Education≥ 14 years	11 (39%)	9 (23%)	37 (47%)	43 (39%)	0.065	43 (40%)	17 (26%)	28 (22%)	47 (25%)	0.017
Living alone	22 (79%)	35 (88%)	66 (85%)	92 (83%)	0.788	94 (87%)	54 (82%)	102 (81%)	138 (73%)	0.028
Supplement(s)	9 (32%)	11 (28%)	19 (24%)	28 (25%)	0.870	35 (32%)	18 (27%)	44 (35%)	53 (28%)	0.534
Habitual intake of *Sunomono*	27 (96%)	37 (93%)	69 (89%)	89 (80%)	0.040	99 (92%)	63 (96%)	116 (92%)	181 (96%)	0.374
Habitual intake of pickles	17 (61%)	29 (73%)	42 (54%)	73 (66%)	0.190	59 (55%)	45 (68%)	88 (70%)	134 (71%)	0.030
Energy intake (kcal/d)	2187 (1568–2797)^ab^	1808 (1535–2147)^a^	2151 (1689–2505)^ab^	2162 (1822–2520)^b^	0.024	1605 (1299–1888)^a^	1634 (1380–2013)^ab^	1515 (1302–1958)^a^	1732 (1451–2064)^b^	0.012
Protein (%E)	14.2 (12.8–16.4)^ab^	13.4 (11.8–16.0)^a^	15.2 (13.7–16.9)^b^	15.1 (12.4–16.1)^ab^	0.015	16.3 (14.7–18.2)	16.0 (14.4–18.0)	16.7 (15.1–18.8)	16.7 (14.9–19.0)	0.389
Fat (%E)	24.6 (21.9–28.1)	23.2 (18.4–28.0)	25.3 (22.9–29.3)	25.9 (22.0–30.0)	0.075	29.1 (26.7–33.9)	28.1 (25.9–31.3)	30.0 (26.0–32.9)	28.4 (24.9–32.0)	0.078
Carbohydrate (%E)	50.9 (47.5–61.0)	51.4 (46.1–61.7)	50.7 (46.8–54.5)	50.9 (43.8–56.0)	0.130	52.4 (46.6–55.6)	53.0 (48.7–57.2)	51.4 (46.3–55.9)	52.8 (49.4–56.7)	0.097
Sodium (mg/d)	4254 (3804–5103)	4630 (4138–5055)	4575 (4114–5281)	4590 (4185–5172)	0.139	3925 (3477–4520)	3885 (3521–4283)	4119 (3647–4587)	4070 (3519–4514)	0.132
Potassium (mg/d)	2713 (2065–2947)	2731 (2164–3234)	2840 (2164–3234)	2754 (2241–3323)	0.125	2656 (2341–3109)	2667 (2400–3027)	2803 (2381–3168)	2830 (2430–3249)	0.210
SFA (g/d)	15.9 (12.6–17.6)	14.0 (12.2–17.2)	16.0 (13.4–18.7)	15.5 (12.4–18.4)	0.387	14.6 (13.1–16.7)	14.5 (12.3–16.0)	14.8 (13.1–16.8)	14.3 (12.4–16.3)	0.289
PUFA (g/d)	14.8 (13.0–17.1)	14.3 (12.3–16.7)	15.0 (13.1–17.5)	15.5 (12.5–17.3)	0.553	14.1 (12.2–16.0)	13.4 (12.2–15.3)	14.2 (12.2–15.3)	13.7 (11.9–15.2)	0.176
n-3 PUFA (g/d)	2.9 (2.3–3.3)	2.9 (2.4–3.6)	3.2 (2.7–3.7)	3.0 (2.4–3.7)	0.140	2.9 (2.5–3.5)	2.7 (2.3–3.3)	2.9 (2.5–3.4)	2.8 (2.5–3.3)	0.107
Seaweed (g/d)	12.8 (5.1–25.6)	14.2 (5.6–17.1)	5.1 (2.4–16.8)	6.6 (2.7–14.2)	0.743	12.3 (4.9–24.6)	11.1 (3.9–19.7)	12.3 (5.3–20.3)	11.1 (4.4–22.2)	0.293
Alcohol (g/d)	8.7 (0.2–28.1)	12.5 (0.0–48.1)	14.4 (0.0–34.6)	17.6 (1.3–42.2)	0.380	0.0 (0.0–1.1)	0.0 (0.0–1.3)	0.0 (0.0–1.0)	0.0 (0.0–0.2)	0.364

Data were presented as mean ± SD or median (interquartile range) for continuous values and number (%) for categorical variables. P values were assessed using ANOVA analyses for continuous variables and a chi-square test for categorical variables. Significant differences among groups were evaluated using Steel-Dwass test. Values with a different superscript letter are significantly different among groups (p < 0.05). DBP, Diastolic blood pressure; SBP, Systolic blood pressure; BMI, body mass index; SFA, saturated fatty acids; PUFA, poly unsaturated fatty acids.

**Figure 2:**
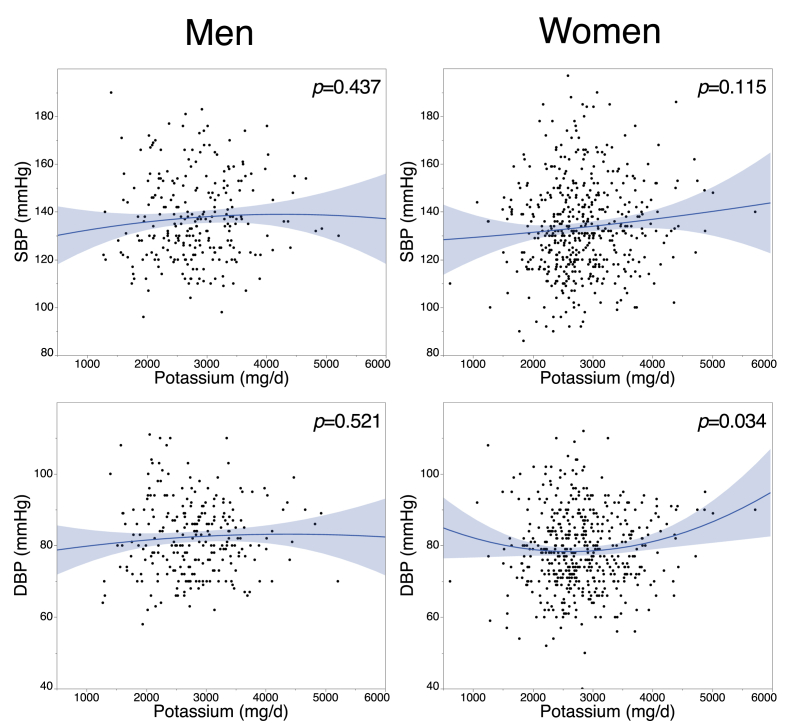
Scatterplot of blood pressure (SBP and DBP) and potassium intake. Data were fitted with a quadratic equation. The shaded region represents the 95% CI.

The relationship between nutrient intake and blood pressure is described in Table 4. The intake of sodium and potassium did not show a significantly strong relationship to SBP or DBP. Potassium intake showed a weak positive relationship to SBP. Application of a quadratic equation revealed that potassium intake and SBP showed U-shape association (Figure 2). Comparing nutrient intake and blood pressure categories (Table 3), energy intake and protein%E in men and energy intake in women were significantly different. However, according to the Pearson's χ^2^-test, energy intake in men did not show significant association with SBP (r = 0.11, p = 0.07) or DBP (r = 0.07, p = 0.27). In men, protein %E in group II was low compared to other groups; however, there was no correlation between protein%E and SBP or DBP. SBP and DBP showed significant negative relationships with carbohydrate%E in men (Table 3). However, compared to the blood pressure categories and the carbohydrate%E did not recognized the significant relationship (Table 3). Other nutrients also failed to show a significant relationship with blood pressure categories.

**Table 4:** The correlation efficient between nutrient intake and blood pressure determined by Pearson's chi-square test.

	Men (n = 257)		Women (n = 489)	
	SBP	DBP	SBP	DBP
Energy intake	0.11 (0.07)	0.07 (0.27)	0.09 (0.04)	−0.01 (0.78)
Protein %E	0.04 (0.53)	0.05 (0.43)	0.06 (0.177)	−0.01 (0.91)
Fat %E	0.05 (0.38))	0.07 (0.24)	−0.09 (0.06)	−0.07 (0.11)
Carbohydrate %E	−0.13 (0.04)	−0.15 (0.02)	0.05 (0.28)	0.05 (0.25)
Sodium	0.09 (0.16)	0.03 (0.59)	0.05 (0.32)	0.02 (0.65)
Potassium	0.07 (0.27)	0.06 (0.32)	0.06 (0.04)	0.07 (0.15)
SFA	0.02 (0.78)	0.06 (0.33)	−0.09 (0.06)	−0.08 (0.07)
PUFA	0.03 (0.67)	0.04 (0.47)	−0.07 (0.12)	−0.06 (0.21)
n-3 PUFA	0.04 (0.48)	0.08 (0.23)	0.04 (0.30)	−0.05 (0.27)
Sea weed	0.00 (0.35)	0.00 (0.94)	0.11 (0.01)	0.02 (0.59)

Data were presented as r (p value). SFA, saturated fatty acids; PUFA, poly unsaturated fatty acids.

We examined the relationship between the intake frequency of vinegared dishes and the blood pressure category. A significant relationship between the frequency of vinegared dishes and blood pressure categories could not be identified in men (data not shown). In contrast, a significant positive-relationship between the frequency of vinegar intake and blood pressure was observed in women (p = 0.014). The results showed that the blood pressure category worsened as the frequency of vinegar consumption increased in women. Interestingly, we noticed that the percentage of blood pressure Group IV was high in the “never” intake group for *Sunomono* compared to other groups in men. Therefore, we evaluated the relationship between blood pressure categories and *Sunomono* intake, not by the intake frequency but by whether *Sunomono* was consumed in a month. Participants who consumed *Sunomono* at least once per month were classified as habitual, whereas those who did not were classified as non-habitual.

Most participants had a habitual intake of *Sunomono*; however, 14% of men and 6% of women did not (Table 2). The percentage of participants who ate pickles was less than that of those that ate *Sunomono*. The percentage of participants who ate *Sunomono* was significantly different among the blood pressure category categories (Table 3). The percentage decreased as the blood pressure category increased. These tendencies were not observed in women. Pickles also contain vinegar. However, there was no relationship between eating pickles and blood pressure category in men. In women, the percentage increased with the blood pressure category (Table 3). Consumption of pickles was significantly related to age in both men and women. The younger the age, the lower the pickle intake. This relationship was more pronounced in women. Therefore, the lower blood pressure category and higher pickle intake in women could be related to age.

We compared the characteristics of the participants based on their intake of *Sunomono* (Table 5). There was no significant difference in age between the two groups in men, but women who did not consume *Sunomono* were significantly younger. SBP and DBP were significantly lower in the habitual group than in the non-habitual group in men. In men, BMI and smoking were comparable between the two groups. The percentage of participants who did not have cohabitants was significantly lower in the non-habitual group. The energy intake of the non-habitual group was 88% of that of the habitual group, and the difference was significant in men. Protein%E and potassium intake also tended to be lower in the non-habitual group. The percentages in the non-habitual group were 89% and 90% for protein%E and potassium, respectively, compared to the habitual group. This tendency was also observed in women. Energy intake, protein %E, and potassium of the non-habitual group were 92%, 90%, and 90%, respectively, compared to the habitual group.

**Table 5:** Characteristics of participants between habitual or non-habitual of *Sunomono*.

Habitual intake of *Sunomono*	Men			Women		
	Yes (n = 222)	No (n = 35)	*p*	Yes (n = 459)	No (n = 30)	*p*
Age (y)	66 (58–73)	65 (59–71)	0.855	66 (60–72)	56 (43–70)	0.002
SBP (mmHg)	134 (123–148)	144 (129–156)	0.027	132 (120–146)	131 (116–140)	0.134
DBP (mmHg)	81 (74–88)	87 (80–94)	0.003	78 (72–87)	78 (70–84)	0.388
BMI (kg/m^2^)	23.1 (21.5–24.9)	24.2 (21.2–25.0)	0.353	22.6 (20.9–24.9)	21.7 (20.4–24.1)	0.134
Smoking Never	62 (28%)	7 (20%)		421 (92%)	24 (80%)	
Former	113 (51%)	21 (60%)	0.536	27 (6%)	5 (17%)	0.127
Current	47 (21%)	7 (20%)		11 (2%)	1 (3%)	
Education ≥ college	83 (37%)	17 (49%)	0.212	123 (27%)	12 (40%)	0.130
Living alone	190 (86%)	25 (71%)	0.048	361 (79%)	27 (90%)	0.108
Supplement(s)	59 (27%)	8 (23%)	0.637	144 (31%)	6 (20%)	0.174
Energy intake (kcal/d)	2143 (1754–2520)	1892 (1458–2301)	0.018	1648 (1354–1971)	1515 (1028–1845)	0.021
Protein (%E)	15.0 (13.0–16.5)	13.4 (12.2–15.3)	0.081	16.6 (15.0–18.7)	14.9 (13.3–18.1)	0.023
Fat (%E)	25.2 (22.0–28.9)	23.8 (18.7–30.1)	0.278	28.9 (25.8–32.4)	28.9 (25.2–33.7)	0.907
Carbohydrate (%E)	50.7 (46.0–56.2)	50.8 (45.4–56.2)	0.857	52.3 (47.8–56.2)	54.7 (48.5–58.9)	0.211
Sodium (mg/d)	4611 (4125–5215)	4399 (4058–4730)	0.071	4047 (3552–4507)	3951 (3467–4558)	0.605
Potassium (mg/d)	2811 (2348–3325)	2538 (2105–3032)	0.058	2727 (2406–3198)	2458 (1928–3012)	0.008
SFA (g/d)	15.6 (13.0–18.1)	14.5 (11.8–18.5)	0.641	14.5 (12.8–16.6)	15.1 (13.6–17.3)	0.187
PUFA (g/d)	15.0 (13.0–17.2)	14.6 (12.1–17.2)	0.318	13.9 (12.2–15.5)	13.5 (11.9–14.8)	0.426
n-3 PUFA (g/d)	3.0 (2.5–3.7)	3.0 (2.4–3.5)	0.411	2.9 (2.5–3.4)	2.6 (2.3–3.0)	0.030
Seaweed (g/d)	14.2 (5.1–19.2)	2.9 (0–6.3)	<0.001	12.3 (4.9–24.6)	3.1 (0–12.3)	<0.001
Alcohol (g/d)	10.9 (0.5–37.1)	21.9 (0.8–42.2)	0.376	0.0 (0.0–0.6)	0.0 (0.0–0.8)	0.686

Data were presented as median (interquartile range) for continuous values and number (%) for categorical variables. P values were assessed using ANOVA analyses for continuous variables and a chi-square test for categorical variables. Significant differences among groups were evaluated using Steel-Dwass test. Values with a different superscript letter are significantly different among groups (p < 0.05). DBP, Diastolic blood pressure; SBP, Systolic blood pressure; BMI, body mass index.

Ordinal logistic analysis was performed to confirm the correlation between the intake of *Sunomono* and the four blood pressure categories. Age, BMI, smoking, overdrinking (≥20 g alcohol/d), energy intake, sodium intake, and potassium intake were selected as factors that are well known to be associated with blood pressure. Energy intake, protein%E, sodium intake, potassium intake, and seaweed intake were categorized into quartiles. Living status was also a factor because it appeared to be closely related to dietary style; indeed, the percentage of men living alone was significantly lower in the non-habitual group (Table 5). In univariate analysis, the intake of *Sunomono* was significantly related to blood pressure in men but not in women (Table 6), and the estimate for *Sunomono* intake was the largest factor. Age was positively related to blood pressure categories in men and women. In women, BMI and living alone were positively and negatively correlated with blood pressure categories, respectively. In multivariate analysis, age, BMI, smoking status, living alone, alcohol consumption, energy intake, protein intake%E, sodium intake, and potassium intake were added as covariates in men. Because of the small number of smokers and drinkers participating, we did not include these as covariates in women. As a result, the estimate for intake of *Sunomono* increased in men. Age and BMI were significant factors for blood pressure categories in women. Using individual SBP and DBP values, we also tried to establish a relationship between blood pressure and vinegar intake with the same method (Table 7). SBP and DBP were negatively associated with *Sunomono* intake in men.

**Table 6:** Ordinal logistic regression analysis of blood pressure categories.

	Men						Women					
	Univariate analysis			Multivariate analysis^$^			Univariate analysis			Multivariate analysis^€^		
	Estimate	95% CI	*p*	Estimate	95% CI	*p*	Estimate	95% CI	*p*	Estimate	95% CI	*p*
Habitual intake of *Sunomono*	−0.489	−0.858, −0.143	0.008	−0.702	−1.122, −0.310	<0.001	0.208	−0.122, 0.540	0.219	−0.073	−0.436, 0.288	0.695
Age	0.035	0.016, 0.054	<0.001	0.033	0.011, 0.056	0.003	0.053	0.037, 0.069	<0.001	0.045	0.026, 0.064	<0.001
BMI	0.027	−0.045, 0.100	0.449	0.030	−0.050, 0.111	0.440	0.103	0.055, 0.151	<0.001	0.112	0.061, 0.164	<0.001
Smoking (former)	0.052	−0.250, 0.354	0.736	−0.095	−0.429, 0.236	0.569	-	-	-	-	-	-
Smoking (current)	−0.234	−0.611, 0.144	0.214	0.019	−0.405, 0.447	0.927	-	-	-	-	-	-
Living alone	−0.015	−0.328, 0.292	0.922	0.087	−0.256, 0.426	0.614	−0.307	−0.513, −0.104	0.003	−0.121	−0.350, 0.107	0.300
Overdrink of alcohol	0.258	−0.028, 0.553	0.078	0.271	−0.040, 0.592	0.087	-	-	-	-	-	-
Energy intake II	0.122	−0.249, 0.540	0.477	0.118	−0.315, 0.557	0.589	−0.198	−0.471, 0.077	0.165	−0.110	−0.400, 0.178	0.458
III	0.268	−0.120, 0.664	0.182	0.274	−0.144 0.699	0.203	0.198	−0.091, 0.490	0.167	0.241	−0.066, 0.550	0.113
IV	0.122	−0.277, 0.527	0.477	0.080	−0.400, 0.566	0.738	0.212	−0.067, 0.494	0.138	0.081	−0.233, 0.400	0.614
Protein %E II	−0.203	−0.587, 0.184	0.306	−0.153	−0.588, 0.283	0.489	−0.041	−0.321, 0.241	0.774	0.029	−0.281, 0.340	0.855
III	0.271	−0.120, 0.669	0.178	0.168	−0.290, 0.631	0.469	−0.200	−0.477, 0.077	0.158	−0.281	−0.584, 0.022	0.072
IV	−0.203	−0.326, 0.449	0.768	−0.125	−0.650, 0.400	0.638	0.378	0.097, 0.663	0.009	0.156	−0.222, 0.537	0.414
Sodium intake II	0.168	−0.221, 0.565	0.401	0.051	−0.370, 0.477	0.816	−0.172	−0.448, 0.104	0.225	−0.096	−0.389, 0.197	0.522
III	0.049	−0.342, 0.445	0.806	0.062	−0.362, 0.492	0.773	0.157	−0.120, 0.436	0.271	0.165	−0.129, 0.460	0.275
IV	0.083	−0.221, 0.565	0.678	0.065	−0.412, 0.545	0.789	0.064	−0.216, 0.345	0.656	−0.052	−0.381, 0.276	0.747
Potassium intake II	−0.001	−0.392, 0.395	0.997	0.008	−0.430, 0.452	0.969	−0.286	−0.564, −0.008	0.044	−0.246	−0.547, 0.055	0.110
III	−0.180	−0.563, 0.204	0.360	−0.270	−0.720, 0.181	0.239	0.182	−0.097, 0.462	0.205	0.222	−0.088, 0.535	0.160
IV	0.259	−0.392, 0.394	0.200	0.266	−0.258, 0.793	0.318	0.228	−0.053, 0.512	0.113	0.064	−0.294, 0.424	0.727
Seaweed intake II	0.255	−0.138, 0.656	0.204	0.333	−0.094, 0.770	0.129	−0.302	−0.570, −0.035	0.027	−0.254	−0.538, 0.059	0.080
III	−0.008	−0.380, 0.367	0.968	0.076	−0.337, 0.493	0.718	0.057	−0.216, 0.331	0.968	−0.001	−0.300, 0.299	0.994
IV	0.162	−0.223, 0.553	0.416	−0.010	−0.500, 0.484	0.968	0.164	−0.224, 0.553	0.416	−0.040	−0.370, 0.292	0.809

^$^Adjusted for age, BMI, smoking history, excessive alcohol intake, living alone, energy intake, protein intake, sodium intake, potassium intake, seaweed intake, ^€^Adjusted for age, BMI, living alone, energy intake, protein intake, sodium intake, potassium intake, seaweed intake.

**Table 7:** The relation of blood pressure and Habitual intake of *Sunomono*.

Men							
SBP				DBP			
Crude Estimate (95% CI)	*p*	Adjusted^$^ Estimate (95% CI)	*p*	Crude Estimate (95% CI)	*p*	Adjusted^$^ Estimate (95% CI)	*p*
−3.87 (−7.13, −0.58)	0.021	−4.08 (−7.46, −0.70)	0.018	−2.96 (−4.80, −1.13)	0.002	−3.16 (−5.19, −1.13)	0.002
Women							
SBP				DBP			
Crude Estimate (95% CI)	*p*	Adjusted^€^ Estimate (95% CI)	*p*	Crude Estimate (95% CI)	*p*	Adjusted^€^ Estimate (95% CI)	*p*
2.58 (−1.00, 6.17)	0.158	0.10 (−3.42, 3.63)	0.95	0.81 (−1.27, 2.89)	0.45	0.99 (−1.19, 3.17)	0.37

Ordinary least squares method to confirm liner regression. ^$^Adjusted for age, BMI, smoking history, excessive alcohol intake, living alone, energy intake, protein intake, sodium intake, potassium intake, seaweed intake, ^€^Adjusted for age, BMI, living alone, energy intake, protein intake, sodium intake, potassium intake, seaweed intake.

## 4 Discussion

Age was a potent factor in the increase in blood pressure categories. The reasons for this have been well described [12]. This relationship was also observed in the present study. Hypertension is associated with obesity [13], defined as a BMI ≥25 kg/m^2^ in Japan. A significant relationship was detected between blood pressure categories and BMI in women. In the present study, we could not find any significant relationship between smoking and blood pressure categories in men. Previous reports have also shown that the relationship between smoking and hypertension is not always detected in cross-sectional studies [14, 15]. However, smoking should be discontinued to help prevent cardiovascular diseases [11].

Vinegar intake was assumed to be related to blood pressure. Therefore, we focused on *Sunomono*, one of the food forms that contributes most to vinegar intake. Ordinal logistic regression analysis indicated that the intake of *Sunomono* was related to blood pressure categories in men. However, we detected that no clear correlation between the frequency of *Sunomono* intake and blood pressure categories. This result suggests that vinegar itself in *Sunomono* is not related to blood pressure. A previous report showed that 30 mL/d of vinegar intake might effectively lower blood pressure, whereas 15 mL/d vinegar intake did not influence blood pressure [8]. Generally, one serving volume of vinegar in *Sunomono* is approximately 10 mL. In the present study, the percentage of participants who consumed vinegar more than twice daily was less than 10%. Therefore, most participants probably did not consume 15 mL of vinegar daily with *Sunomono*.

*Sunomono* is a seaweed (*Mozuku* or *Mekabu*) and/or cucumber seasoned with soy sauce and vinegar. In Japanese supermarkets, *Sunomono* with seaweed is sold in packs (60–80 g/pack) to make it easier for consumers to buy. Therefore, it was expected that there would be a correlation between the consumption of *Sunomono* and seaweed. Indeed, seaweed consumption was significantly higher in the habitual intake *Sunomono* group (Table 5). Seaweed is a rich source of potassium, which reduces SBP and DBP in people with hypertension [16]. However, potassium intake did not show a significant relationship with SBP and DBP categories in the present study.

Pickles are also vinegar-related food. However, the intake habits for pickles were not related to blood pressure categories. In Japan, the pickles that were mainly consumed were pickled *Allium chinense* called *Rakkyo*. The intake volume of vinegar may be less from pickled *Rakkyo* than from *Sunomono* because the liquid with pickled *Rakkyo* is not usually served. We concluded that the intake habits of pickled *Rakkyo* did not affect blood pressure at the side dish serving level.

We sought to determine whether the intake of *Sunomono* might be related to lifestyle habits related to blood pressure, but we did not find any significant factors. We propose that the combination of seaweed and vinegar intake has a beneficial effect on blood pressure. Although it is not currently possible to explain why even less frequent intake of *Sunomono* affects blood pressure, one possibility is that there is improvement in the intestinal environment because of the intake of seaweed and vinegar. Human studies have shown that the alpha diversity of gut microbiota is related to blood pressure [17]. Briefly, gram-negative microbiota are highly abundant in the high blood pressure category, whereas short-chain fatty acid (SCFA)-producing bacteria are less abundant compared to that of normotensive patients. Recently, Zhu et al. reported the effect of vinegar intake on gut microbiota using a rat model of hyperoxaluria [18]. They showed that the abundance of SCFA-producing bacteria was significantly increased by daily oral administration of 2 mL/kg Shanxi-aged vinegar containing water for 4 weeks, but there was a concern regarding the extremely high vinegar dosage. Additionally, 16-week seaweed supplementation also increased the abundance of SCFA-producing bacteria in rats. It has not been reported whether their combination enhances the respective effects of vinegar and seaweed on gut microbiota or blood pressure. Further research is needed to clarify the mechanism by which weekly *Sunomono* consumption has a blood pressure-lowering effect.

The present findings should be evaluated within the limitations of this study. First, the cross-sectional design of the study did not facilitate causal inferences. Future longitudinal studies are required to accumulate adequate evidence in this field. Second, the all portion size of vinegared dishes has not been verified and we did not measure how the participants consumed vinegar liquid with *Sunomono* or pickles to evaluate the consumption volume of vinegar. It would be difficult to precisely estimate the consumption of the picked liquid using the questionnaire. We considered the results of intake frequency as intake habits and used them in the analysis. Third, the proportion of the non-intake habits of side dishes containing vinegar was low. Fourth, the present conclusions are limited to men. There were several possible reasons: 1) the population with non-intake habits for side dishes containing vinegar in women was lower than that in men; 2) SBP and DBP were lower in women than men; 3) the age of the non-intake habitual group in women was quite young in women. Fifth, the BDHQ is a self-reported questionnaire. Therefore, random and systematic measurement errors could impact the results of the BDHQ, and this effect has been seen in other self-reported dietary assessment methods. To minimize the effect of misreporting, we excluded participants who reported low or high energy intake and used energy-adjusted values. Sixth, we could not explain the mechanism by which the non-intake habit of side dishes containing vinegar was related to worsened blood pressure categories. Non-intake habitual participants may have a lifestyle that leads to high blood pressure. Exercise habits are one of the lifestyle factors. However, exercise habits were not related to blood pressure or dietary habits of *Sunomono* (data not shown). Finally, residual confounding factors may occur in every observational study; therefore, our results need to be confirmed by an intervention study on the population with the non-intake habit of side dishes containing vinegar.

Nonetheless, our study had several strengths and novel features. Many nutrients and background factors related to blood pressure were analyzed simultaneously. To the best of our knowledge, this is the first report on the relationship between intake of *Sunomono* and blood pressure.

In conclusion, the intake of *Sunomono*, but not *Rakkyo*, was significantly and independently associated with blood pressure categories in men. The intake of *Sunomono* could help improve the blood pressure categories.

## Declarations

### Author contribution statement

Hiroaki Kanouchi: Conceived and designed the experiments; Performed the experiments; Wrote the paper.

Mikako Yamashita, Kaori Kaimoto: Performed the experiments; Analyzed and interpreted the data.

Akiko Kuwabara, Yukiko Kawakami, Shigeo Takenaka: Analyzed and interpreted the data.

Chihaya Koriyama, So Kuwahata, Toshihiro Takenaka, Yuichi Akasaki, Masaaki Miyata: Performed the experiments.

Takuro Kubozono: Conceived and designed the experiments; Performed the experiments.

Mitsuru Ohishi: Conceived and designed the experiments; Performed the experiments; Contributed reagents, materials, analysis tools or data.

### Funding statement

This research did not receive any specific grant from funding agencies in the public, commercial, or not-for-profit sectors.

### Data availability statement

The authors do not have permission to share data.

### Declaration of interest's statement

The authors declare no conflict of interest.

### Additional information

No additional information is available for this paper.
